# Complete Sequence and Comparative Analysis of the Chloroplast Genome of Coconut Palm (*Cocos nucifera*)

**DOI:** 10.1371/journal.pone.0074736

**Published:** 2013-08-30

**Authors:** Ya-Yi Huang, Antonius J. M. Matzke, Marjori Matzke

**Affiliations:** Institute of Plant and Microbial Biology, Academia Sinica, Taipei, Taiwan; Universidad Miguel Hernández de Elche, Spain

## Abstract

Coconut, a member of the palm family (Arecaceae), is one of the most economically important trees used by mankind. Despite its diverse morphology, coconut is recognized taxonomically as only a single species (*Cocos nucifera* L.). There are two major coconut varieties, tall and dwarf, the latter of which displays traits resulting from selection by humans. We report here the complete chloroplast (cp) genome of a dwarf coconut plant, and describe the gene content and organization, inverted repeat fluctuations, repeated sequence structure, and occurrence of RNA editing. Phylogenetic relationships of monocots were inferred based on 47 chloroplast protein-coding genes. Potential nodes for events of gene duplication and pseudogenization related to inverted repeat fluctuation were mapped onto the tree using parsimony criteria. We compare our findings with those from other palm species for which complete cp genome sequences are available.

## Introduction

Chloroplasts (cp) are cell organelles that carry out photosynthesis, thus converting light energy into chemical energy in green plants and algae. Chloroplasts contain their own genome, which in flowering plants usually consists of a circular double-stranded DNA molecule ranging from 120 to 160 kb in length [1]. The cp genome is divided into four parts comprising a large single copy region (LSC) and a small single copy region (SSC), which are separated by a pair of inverted repeats (IRs). Cp genomes typically encode four rRNAs, around 30 tRNAs and up to 80 unique proteins [2]–[4].

With the advent of high-throughput sequencing technologies and their use in obtaining complete plastid genomes [5], [6], the number of fully sequenced cp genomes has increased rapidly. To date, the Complete Organelle Genome Sequences Database (http://amoebidia.bcm.umontreal.ca/pg-gobase/complete_genome/ogmp.html) lists 324 complete cp genome sequences spanning 268 distinct organisms. The complete cp genome sequences include date palm (*Phoenix dactylifera* L.) and oil palm (*Elaeis guineensis* Jacq.). Both are members of the palm family (Arecaceae), which is the third most economically important family of plants after the grasses and legumes [7]. Complete sequence information on cp genomes from three additional palms - *Calamus caryotoides*, *Pseudophoenix vinifera*, *Bismarkia nobilis* – has recently been deposited in GenBank [8]. However, the complete cp genome sequence of coconut palm (*Cocos nucifera* L.), which is a universal symbol of the tropics and equally important as oil palm [7], has not yet been reported.

Coconut is one of the most important crops in tropical zones where it is a source of food, drink, fuel, medicines and construction material [9]. In addition, coconut oil is used for cooking and for pharmaceutical and industrial applications [10]. Although coconut trees display considerable morphological diversity, they are considered taxonomically a single species (and the only species) within the genus *Cocos*. Based on stature and breeding, coconut cultivars can be divided into two groups: tall and dwarf [11]. The former typically grows up to 35 to 40 meters and is mainly outcrossing, whereas the latter can only grow up to 25 to 30 meters and usually is selfing. Dwarf coconuts, which are less common than the tall variety, are usually found growing close to humans and have traits that likely result from human selection [10]. Here we report the complete cp genome sequence of a dwarf coconut plant, which is thought to be descended from coconut trees originally imported into Taiwan from Thailand (personal communication from private breeder).

## Materials and Methods

### Whole genome sequencing and de novo assembly

Fresh young leaf material (ca. 2 g) was collected from a coconut seedling growing under ambient conditions in the greenhouse of Academia Sinica and the genomic DNA (gDNA) was extracted using a modified CTAB protocol [12]. We used the ratio of absorbance at 260 nm and 280 nm (A260/280) and gel electrophoresis to measure the purity and integrity of the extracted gDNA. High quality DNA (concentration >100 ng/µl; A260/230>1.7; A260/280 = 1.8∼2.0) was sequenced using the Illumina GAIIx platform (YOURGENE BIO SCIENCE Co., New Taipei City, Taiwan). Short reads (70 bp) from paired-end sequencing were trimmed with a 0.05 error probability. The trimmed reads were *de novo* assembled using CLC Genomic Workbench 6.0.1 (CLC Bio, Aarhus, Denmark). The de Bruijn Graph approach with a k-mer length of 22 bp and a coverage cutoff value of 10X was applied for assembly. The average read length and insert size were 151 bp and 340 bp respectively. The assembled contigs shorter than 200 bp were removed from the scaffold while those with coverage larger than 10X were selected for BLAST search against plastid genomes of date palm [2], oil palm [3], and other chloroplast sequences with an e-value cutoff of 10^−5^ (199 sequences in total). Gaps between contigs were filled by PCR amplification with specific primers that were designed based on contig sequences or homologous sequence alignments (Table S1). The PCR products were purified with GEL/PCR DNA clean-up kit (Favorgen Biotech Corp.) and then sequenced by conventional Sanger sequencing. The sequencing data along with gene annotation have been submitted to GenBank with an Accession number of KF285453.

### Genome annotation, base composition, repeat structure, and codon usage

Preliminarily gene annotation was carried out through the online program DOGMA [13] and BLAST searches. To verify the exact gene and exon boundaries, we used MUSCLE [14] to align putative gene sequences with their homologues acquired from BLAST searches in GenBank. All tRNA genes were further confirmed through online tRNAscan-SE search server [15]. The online program tandem repeat finder [16] was used to search the locations of repeat sequences (>10 bp in length) with the following set up: (2, 7, 7) for alignment parameters (match, mismatch, indels); 80 for minimum alignment score to report repeat; and maximum period size of 500. Codon usage was calculated for all exons of protein-coding genes (pseudogenes were not calculated). Base composition was calculated by Artemis [17].

### Analysis of RNA editing

Potential RNA editing sites in protein-coding genes of coconut cpDNA were predicted by the online program Predictive RNA Editor for Plants (PREP) suite (http://prep.unl.edu/) [18] with a cutoff value of 0.8. This program contains 35 reference genes for detecting RNA editing sites in plastid genomes. The predicted editing sites were verified by reverse transcription polymerase chain reaction (RT-PCR) experiments. In addition to those genes predicted by the program, we also investigated *rpl22*, *rpl23*, *rps3*, *rps7*, *ycf1*, *ycf2*, and *ycf4* genes, within which RNA editing sites were reported in the cp genome of oil palm [3]. The Plant Total RNA Miniprep Purification Kit (GMbiolab Co., Ltd.) was applied to extract total RNA from leaf of the same seedling used for DNA extraction. The first strand cDNA was synthesized with QuantiTect Reverse Transcription Kit (Qiagen) following the manufacturer's protocol. Gene specific primers for cDNA amplification were designed based on homologous sequence alignment. Maximum 1 µl of the reaction mixture was used as template for PCR amplification. The PCR products were purified with GEL/PCR DNA clean-up kit (Favorgen Biotech Corp.). Purified PCR products were sequenced using ABI PRISM® 3700. A complete primer list is provided in Table S1.

### Phylogenetic analysis

Forty seven protein coding genes were extracted from 25 taxa, including *Amborella*, *Nuphar*, 17 species of monocots, four species of magnoliids, and two species of eudicots. The GenBank accession number of each taxon is provided in Table 1. These taxa were selected because they have complete or nearly complete plastid genomes deposited in GenBank. Nucleotide sequences of each gene were first aligned by MUSCLE [14] through the online server of European Bioinformatics Institute (http://www.ebi.ac.uk/Tools/msa/muscle). The aligned sequences were then concatenated through copy and paste in text editor. The statistical method of Maximum Likelihood (ML) and the computer program Garli version 2.0 were applied for phylogenetic reconstruction, with parameters estimated from the data. The GTR substitution model with evolutionary rates among sites evaluated by a discrete gamma distribution was used for tree search. All positions containing gaps or missing data were eliminated. Branch support was evaluated by 1,000 replications of bootstrap (BS) re-sampling.

**Table 1:** Accessions and references for taxa used in phylogenetic reconstruction and genome comparison in this study.

Taxon	GenBank accession number	Reference
Basal angiosperms		
*Amborella trichopoda*	NC_005086	Goremykin et al. 2003 [25]
*Nuphar advena*	NC_008788	Raubeson et al. 2007 [26]
Monocots		
*Acorus americanus*	EU273602	Unpublished
*Colocasia esculenta*	JN105690	Ahmed et al. 2012 [28]
*Cymbidium aloifolium*	KC876122	Yang et al. 2013 [29]
*Bismarckia nobilis*	JX088664	Barrett et al. 2013 [8]
*Calamus caryotoides*	JX088663	Barrett et al. 2013 [8]
*Chamaedorea seifrizii*	JX088667	Barrett et al. [8]
*Cocos nucifera*	KF285453	Produced in this study
*Elaeis guineensis*	JF274081	Uthaipasanwong et al. 2012 [3]
*Phoenix dactylifera*	GU811709	Yang et al. 2010 [2]
*Pseudophoenix vinifera*	JX088662	Barrett et al. 2013 [8]
*Dasypogon bromeliifolius*	JX088665	Barrett et al. 2013 [8]
*Kingia australis*	JX051651	Barrett et al. 2013 [8]
*Typha latifolia*	GU195652	Jansen et al. 2007 [30]
*Alpinia zerumbet*	JX088668	Barrett et al. 2013 [8]
*Heliconia collinsiana*	JX088660	Barrett et al. 2013 [8]
*Musa acuminata*	HF677508	Martin et al. 2013 [59]
*Xiphidium caeruleum*	JX088669	Barrett et al. 2013 [8]
Magnoliids		
*Chloranthus spicatus*	EF380352	Hansen et al. 2007 [31]
*Drimys granadensis*	DQ887676	Cai et al. 2006 [5]
*Magnolia denudata*	JN867577	Unpublished
*Piper cenocladum*	DQ887677	Cai et al. 2006 [5]
Eudicots		
*Ceratophyllum demersum*	NC009962	Moore et al. 2007 [27]
*Nandina demostica*	DQ923117	Moore et al. 2006 [32]

## Results and Discussion

### Sequencing and de novo assembly

Illumina sequencing produced 6,413,504 paired-end reads with an average read length of 151 bp and a total base number of 968,439,104. After quality trim, 6,328,120 reads with an average of 145.3 bp and a total base number of 919,475,836 remain. The subsequent *de novo* assembly and reference-guided blast search resulted in five major contigs separated by five gaps, which were then filled by Sanger sequencing. In addition to gap closure and confirmation of four junction regions (LSC/IR_A_, LSC/IR_B_, SSC/IR_A_, SSC/IR_B_), we also validated the accuracy of our whole genome sequencing by randomly selecting genes/spacers for PCR-based sequencing. Priority was given to long genes (e.g., *ycf1*, *ycf2*, *rpoC1*) or long spacers (between pairs of *rpoB* and *psbD*, *ycf2*and*ndhB*, *ndhC* and *trnV-UAC*). A few regions where genes were transcribed from clockwise to counterclockwise (vice versa) were also validated.

### Organization of chloroplast genome

**Figure 1:**
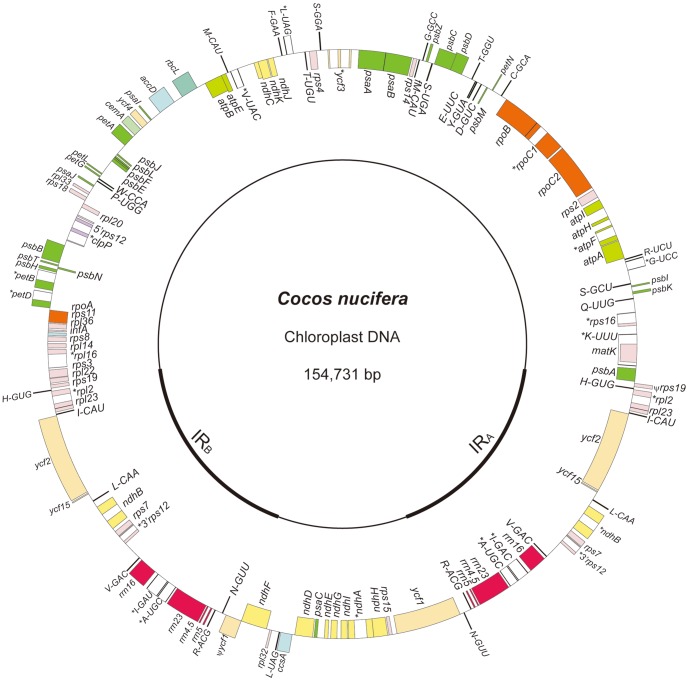
Coconut chloroplast genome map. Genes shown on the outside of the large circle are transcribed clockwise, while genes shown on the inside are transcribed counterclockwise. Thick lines of the small circle indicate IRs. Genes with intron are marked with “*”. Pseudo genes are marked with “Ψ”.

Analysis of the data obtained from high-throughput sequencing demonstrated that the cp genome of coconut is a typical quadripartite molecule (Fig. 1) within which a pair of inverted repeats (IRs) is separated by a large single copy region (LSC) and a small single copy region (SSC). The genome is 154,731 bp in length (IRs = 53,110 bp; LSC = 84,230 bp; SSC = 17,391 bp) and is predicted to encode 130 genes and four pseudogenes. The former includes 84 protein-coding genes, 38 tRNA genes, and eight rRNA genes while the latter is represented by pseudo *ycf1*, *rps19*, and two copies of *ycf15*. Of those genes, three protein-coding genes (*ycf2*, *ndhB*, and *rps7*), four rRNA genes (*rrn16*, *rrn23*, *rrn4.5*, and *rrn5*), and eight tRNA genes are present in two copies (Fig. 1).

Fourteen of the protein-coding genes and eight of the tRNA genes contain introns; and four pairs of genes overlap (4 bp between *atpE* and *atpB*; 10 bp between *ndhK* and *ndhC*; 53 bp between *psbC* and *psbD*; and 57 bp between pseudo *ycf1* and *ndhF*). Each intron-containing gene has only one intron, except *ycf3* and *clpP*, which have two introns. Most protein-coding genes have standard AUG as initiator codon; however, *rpl2* and *ndhD* have an initiator codon of ACG, *rps19* starts with a GUG codon, and the initiator codon of *cemA* is ambiguous. The frequency of codon usage in the coconut cp genome is summarized in Table 2. Similar to many cp genomes of angiosperms [2], [3], [19]–[22], a strong bias toward an A or T in the third position of synonymous codons is also observed in the coconut cp genome. The most and least prevalent amino acids are leucine (2624) and cysteine (323), respectively.

**Table 2:** Codon usage and codon-anticodon recognition pattern in cp genome of coconut.

Amino acid	Codon	No	RSCU	tRNA	Amino acid	Codon	No	RSCU	tRNA
Phe	UUU	906	1.23		Ala	GCA	383	0.59	*trnA-UGC*
	UUC	564	0.77	*trnF-GAA*		GCC	202	0.31	
Leu	UUA	785	0.60			GCG	123	0.19	
	UUG	556	0.42	*trnL-CAA*		GCU	586	0.91	
	CUA	371	0.28	*trnL-UAG*	Tyr	UAU	769	1.59	
	CUC	188	0.14			UAC	196	0.41	*trnY-GUA*
	CUG	173	0.13		His	CAC	144	0.45	*trnH-GUG*
	CUU	551	0.42			CAU	493	1.55	
Ile	AUA	718	0.64	*trnI-CAU*	Gln	CAA	668	1.49	*trnQ-UUG*
	AUC	487	0.43	*trnI-GAU*		CAG	226	0.51	
	AUU	1045	0.93		Asn	AAC	274	0.44	*trnN-GUU*
Met	ATG	613	1.00	*trn(f)M-CAU*		AAU	967	1.56	
Val	GUA	517	0.74	*trnV-UAC*	Lys	AAG	353	0.53	
	GUC	188	0.27	*trnV-GAC*		AAA	988	1.47	*trnK-UUU*
	GUG	190	0.27		Asp	GAC	209	0.39	*trnD-GUC*
	GUU	497	0.71			GAU	863	1.61	
Ser	AGC	104	0.10	*trnS-GCU*	Glu	GAA	1009	1.49	*trnE-UUC*
	AGU	414	0.40			GAG	346	0.51	
	UCA	440	0.43	*trnS-UGA*	Cys	UGC	78	0.48	*trnC-GCA*
	UCC	338	0.33	*trnS-GGA*		UGU	245	1.52	
	UCG	179	0.17		Trp	TGG	444	1.00	*trnW-CCA*
	UCU	574	0.56		Arg	AGA	512	0.65	*trnR-UCU*
Pro	CCA	312	0.59	*trnP-UGG*		AGG	161	0.20	
	CCC	207	0.39			CGA	345	0.44	
	CCG	131	0.25			CGC	89	0.11	
	CCU	407	0.77			CGG	123	0.16	
Thr	ACA	417	0.64	*trnT-UGU*		CGU	344	0.44	*trnR-ACG*
	ACC	241	0.37	*trnT-GGU*	Gly	GGA	712	0.83	*trnG-UCC*
	ACG	149	0.23			GGC	143	0.17	*trnG-GCC*
	ACU	504	0.77			GGG	276	0.32	
						GGU	587	0.68	

RSCU: Relative Synonymous Codon Usage.

Although RT-PCR analysis validated that C-to-U editing changed the ACG start codon to AUG in the *ndhD* gene, the ACG start codon in the *rpl2* gene appeared to remain unedited in repeated experiments. However, we cannot eliminate the possibility that a low level of editing occurs in *rpl2*. Although less frequent than AUG, translation initiated at an ACG or GTG start codon is not unprecedented in plants. A previous study demonstrated that an initiator codon of AUG is not required to specify the initiation site for a proper translation in the cp genome [23]. GUG codons have been shown to be more efficient than ACG in initiating translation and have a relative strength varying from 15 to 30% of AUG activity [24]. In angiosperms, a GUG start codon has been found in the *cemA* gene [5], [25]–[27] and *rps19* gene [2], [3], [5], [8], [26], [28]–[32]. A transcript starting with an ACG start codon has been observed in the *ndhD* gene in some species of *Nicotiana* [33], [34].

### Repeats

With a criterion of 100% match in repeat copies, the tandem repeat finder identified 13 sets of repeats that are longer than 10 bp, including eight tandem repeats, three direct repeats, and two inverted repeats (Table 3). Three of the repeats are found in the *ycf2* genes, which are in the IR regions. The remaining repeats are found in the LSC region: one at the 3′ end of the *rps3* gene, seven in spacers, and two in the introns. This repeat content is similar to that found in date palm and oil palm. In fact, five of the repeats found in coconut (No. 2, 3, 6, 11and 12 in Table 3) are shared by both oil palm and date palm, though the copy number may differ. In addition, repeats No. 5 and No. 8 in coconut are shared by oil palm while repeats No. 4 and 13 are shared by date palm.

**Table 3:** Repeat sequences and their distribution in cpDNA of coconut.

No.	Size (bp)	Start position	Repeat number	Type	Repeat sequence	Region
1	30	64504, 64537	2	D	TATACTATAATAAATATACTATAATAAATA	LSC; spacer between *psbE* and *petL*
2	24	91629, 91653, 91677	3	T	GATATCGATATTGATGATAGTGAC	IRB; *ycf2* gene
3	24	146981, 147005, 147029	3	T	ATATCGTCACTATCATCAATATCG	IRA; *ycf2* gene
4	21	149421, 149442	2	T	GAAGTGACTTGGACAAAAAGA	IRA; *ycf2* gene
5	20	31427, 31447	2	T	TTAAAAGATATACTCTGGAA	LSC; spacer between *trnT* and *psbD*
6	20	82734, 82754	2	T	CTCGTTTACAAATATCCAAA	LSC; 3′ end of *rps3* gene
7	19	64518, 64537	2	T	TATACTATAATAAATATAC	LSC; spacer between *psbE* and *petL*
8	17	12731, 12748	2	T	TTCTTTATTTGTATTTG	LSC; intron of *atpF* gene
9	13	28852, 28873	2	D	TATTATATATAAA	LSC; spacer between *petN* and *psbM*
10	13	*59048	1	I	TATTATATATAAA	LSC; spacer between *petN* and *psbM*, spacer between *accD* and *psaI*
11	12	3749, 3773, 3793	3	D	AATTAAATAATA	LSC; intron of *trnK*
12	12	35106, 35118, 35141	3	T	ACTACTATACTA	LSC; spacer between *trnG* and *trnfM*
13	12	**35167	1	I	ACTACTATACTA	LSC; spacer between *trnG* and *trnfM*

D: direct repeat; T: tandem repeat; I: inverted repeat.

*: inverted repeat sequence of repeat No. 9;

**: inverted sequence of repeat No. 12.

Repetitive sequences in cp genomes may recombine and induce rearrangements [35]–[37], which could play a crucial role in stabilization of cpDNA [38]. Compared with other angiosperms, cp genomes of the palm family generally have fewer and shorter repeats (Table 4). Of the 13 repeats found in coconut cpDNA, the longest is 30 bp. The oil palm cp genome has seven repeats and the longest is 40 bp [3] while date palm has 11 repeats and the longest is 39 bp [2]. By contrast, more than 20 repeats, with the longest extending up to 132 bp, were reported in Poaceae [39], [40]. About 232 repeats, ranging from 30 to 61 bp in length, were reported in *Cymbidium* orchid [29]. In *Citrus*, 29 repeats with a range of 30 to 59 bp in length were detected [41]. In the Solanaceae family, as many as 42 repeats, with the most extensive being 56 bp, have been reported [42].The cp genome of *Gossypium*has 54 repeats, with a longest one of 64 bp [43]. In the Geraniaceae family, some cp genomes contain up to 9% (or higher) repetitive DNA [4], [44] and many of the repeats are longer than 100 bp [4].

**Table 4:** Comparison of repeat numbers and repeat lengths among 16 angiosperms.

Taxon	Total repeats	Longest repeat	References
	(No.)	(bp)	
**Monocots**			
Orchidaceae			
*Cymbidium aloifolium*	232	61	Yang et al. 2013 [29]
Arecaceae			
*Cocos nucifera*	13	30	Produced in this study
*Elaeis guineensis*	7	40	Uthaipaisanwong et al. 2012 [3]
*Phoenix dactylifera*	11	39	Yang et al. 2010 [2]
Poaceae			
*Bamboo emeiensis*	39	132	Zhang et al. 2011 [39]
*Hordeum vulgare*	31	>55	Sasaki et al. 2007 [40]
*Sorghum bicolor*	26	>55	Sasaki et al. 2007 [40]
*Agrostis stolonifera*	19	>55	Sasaki et al. 2007 [40]
**Dicots**			
Geraniaceae			
*Geranium palmatum*	100–150	>200	Guisinger et al. 2011 [4]
*Pelargonium hortorum*	ca. 200	>200	Guisinger et al. 2011 [4]
Rutaceae			
*Citrus sinensis*	29	53	Bausher et al. 2006 [41]
Malvaceae			
*Gossypium hirsutum*	54	72	Lee et al. 2006 [43]
Solanaceae			
*Atropa belladonna*	40	45–49	Daniell et al. 2006 [42]
*Nicotiana tabacum*	33	>55	Daniell et al. 2006 [42]
*Solanum lycopersicum*	40	>55	Daniell et al. 2006 [42]
*Solanum tuberosum*	31	50–54	Daniell et al. 2006 [42]

In view of the correlation between repetitive DNA content and sequence rearrangement, significant structural rearrangements are likely to be observed in cp genomes rich in repetitive sequences. This idea has been validated in many cases listed above such as Poaceae [35], [39], [40], [42] and Geraniaceae [4], [44]–[46]. Conversely, the relatively low content of repetitive DNA in cp genomes of the palm family suggests a relatively higher degree of stability and conservation across different palm species. Consistent with this notion, our investigation revealed neither significant recombination (Fig. S1) nor dramatic variation (Table 5) in the cp genomes of six palm species.

**Table 5:** Comparison of cp genomes among six palm species.

Characteristics	*Calamus*	*Pseudophoenix*	*Phoenix*	*Bismarckia*	*Elaeis*	*Cocos*
**Size (bp)**	157,270	157,829	158,462	158,211	156,973	154,731
LSC	85,525	85,736	86,198	86,390	85,192	84,230
SSC	17,595	17,587	17,712	17,459	17,639	17,391
IR	54,150	54,506	54,552	54,362	54,142	53,110
GC content (%)	37.36	37.32	37.23	37.47	37.40	37.44
**Total number of genes**	131	131	131	131	131	129
Protein-coding genes	85	85	85	85	85	84
G+C (%)	38	38	38	38	38	37
bases (bp)	192,481	191,886	192,511	120,079	10,782	90,130
rRNAs	8	8	8	8	8	8
G+C (%)	55	55	55	55	55	55
bases (bp)	9,050	9,051	9,050	9,050	7,040	9,040
tRNAs	38	38	38	38	38	38
G+C (%)	53	53	53	53	53	44
bases (bp)	10,748	10,756	10,766	10,789	10,782	10,570
**Number of Pseudogenes**	1	1	1	1	1	2
**Gene with intron(s)**	22	22	22	22	22	22
Protein-coding genes	14	14	14	14	14	14
tRNAs	8	8	8	8	8	8

### RNA editing sites

RNA editing is a posttranscriptional process that is mainly observed in mitochondrial and cp genomes of higher plants [47]. This process may induce the occurrence of substitution or indels, which in turn, can result in transcript alternation [33], [47], [48]. In coconut cpDNA, the PREP-cp program predicted 83 RNA editing sites out of 27 genes. Our RT-PCR analysis confirmed editing at 64 of those sites (Table 6). An additional six editing sites not predicted by the program were detected in *accD*, *matK*, *ndhB*, *ndhG*, *ndhH*, and *rpoA*. Of the genes investigated, *ndh* genes have the highest number of editing sites.

**Table 6:** RNA editing predicted by PREP-cp program and confirmed by RT-PCR.

Gene	Nucleotide Position	Codon change	Editing position within codon	Amino acid change	PREP Predicted	RT-PCR results
accD	154	CGG - TGG	1	R-W	+	-
	794	TCG - TTG	2	S-L	-	+
	1157	TCA - TTA	2	S-L	+	+
	1159	CAT - TAT	1	H-Y	+	-
	1403	CCT - CTT	2	P-L	+	-
atpA	914	TCA - TTA	2	S-L	+	+
	1148	TCA - TTA	2	S-L	+	+/−
atpB	1184	TCA - TTA	2	S-L	+	+*
atpF	92	CCA - CTA	2	P-L	+	+/−*
atpI	428	CCC - CTC	2	P-L	+	+
	629	TCA - TTA	2	S-L	+	+
ccsA	647	ACT - ATT	2	T-I	+	-
clpP	82	CAT - TAT	1	H-Y	+	+*
	559	CAT - TAT	1	H-Y	+	+*
matK	188	TCA - TTA	2	S-L	+	-
	653	CCA - CTA	2	P-L	+	-
	734	TTC - TTT	3	D- F	-	+
	919	CAT - TAT	1	H-Y	+	-
	1267	CAC - TAC	1	H-Y	+	+
ndhA	50	TCG - TTG	2	S-L	+	+
	476	TCA - TTA	2	S-L	+	+
	566	TCA - TTA	2	S-L	+	+
	961	CCT - TCT	1	P-S	+	+
	1073	TCC - TTC	2	S-F	+	-
ndhB	149	TCA - TTA	2	S-L	+	+/−
	467	CCA - CTA	2	P-L	+	+
	542	ACG - ATG	2	T-M	+	+
	586	CAT - TAT	1	H-Y	+	+
	704	TCC - TTC	2	S-F	+	+
	737	CCA - CTA	2	P-L	-	+*
	830	TCA - TTA	2	S-L	+	+
	836	TCA - TTA	2	S-L	+	+
	1112	TCA - TTA	2	S-L	+	+
	1193	TCA - TTA	2	S-L	+	+
	1255	CAT - TAT	1	H-Y	+	+
	1481	CCA - CTA	2	P-L	+	+/−
ndhD	2	ACG - ATG	2	T-M	+	+/−
	59	TCA - TTA	2	S-L	+	+
	383	TCA - TTA	2	S-L	+	+
	674	TCG - TTG	2	S-L	+	+
	947	ACA - ATA	2	T-I	+	+
	1193	TCA - TTA	2	S-L	+	+
	1310	TCA - TTA	2	S-L	+	+
ndhF	62	TCA - TTA	2	S-L	+	+/−
	290	TCA - TTA	2	S-L	+	+/−
	392	TCC - TTC	2	S-F	+	+
	442	CAT - TAT	1	H-Y	+	+
	586	CTT - TTT	1	L-F	+	-
	1393	CAC - TAC	1	H-Y	+	-
	2093	TCC - TTC	2	S-F	+	-
ndhG	314	ACA - ATA	2	T-I	+	-
	347	CCA - CTA	2	P-L	-	+
ndhH	505	CAT - TAT	1	S-L	+	+
	545	TCT - TTT	2	S-F	-	+/−*
	726	TAC - TAT	3	Y-Y	-	-
ndhK	131	TCG - TTG	2	S-L	+	+
	372	GTC - GTT	3	S-L	-	-
	518	ATG - ACG	2	M-T	-	-
	677	TCA - TTA	2	S-L	-	-
petB	418	CGG - TGG	1	R-W	+	+*
	611	CCA - CTA	2	P-L	+	+
psaI	80	TCT - TTT	2	S-F	+	+
	85	CAT - TAT	1	H-Y	+	+
rpl2	2	ACG - ATG	2	T-M	+	-
rpl20	26	ACA - ATA	2	T-I	+	-
	308	TCA - TTA	2	S-L	+	-
rpl22	242	TCA - TTA	2	S-L	-	-
rpl23	71	TCA - TTA	2	S-L	-	+/-*
	89	TCT - TTT	2	S-F	-	+/-*
rpoA	200	TCT - TTT	2	S-F	-	+
	368	TCA - TTA	2	S-L	+	+
	527	TCC - TTC	2	S-F	+	+
	830	TCA - TTA	2	S-L	+	+
	887	TCG - TTG	2	S-L	+	-
rpoB	467	TCG - TTG	2	S-L	+	+/-
	545	TCA - TTA	2	S-L	+	+
	560	TCG - TTG	2	S-L	+	+
	617	CCG - CTG	2	P-L	+	+/-
	1994	TCT - TTT	2	S-F	+	+
	2420	TCA - TTA	2	S-L	+	+/−
rpoC1	41	CCA - CTA	2	P-L	+	+
	511	CGG - TGG	1	R-W	+	+
	617	TCA - TTA	2	S-L	+	+
	1663	CAT - TAT	1	H-Y	+	-
rpoC2	1381	CAT - TAT	1	H-Y	+	-
	2275	CGG - TGG	1	R-W	+	-
	2309	TCG - TTG	2	S-L	+	+
rps2	134	ACA - ATA	2	T-I	+	+
	248	TCA - TTA	2	S-L	+	+
rps3	30	TTC - TTT	3	I-I	-	-
	470	ACA - ATA	2	S-L	-	+/−*
	583	CAT - TAT	1	S-L	-	+*
	627	ATC - ATT	3	I-I	-	-
rps7	300	GCC - GCT	3	A-A	-	-
rps8	141	AAT - AAC	3	N-N	-	-
	182	TCA - TTA	2	S-L	+	+
rps14	80	TCA - TTA	2	S-L	+	+
	149	CCA - CTA	2	P-L	+	+
ycf1	3423	TAC - TAT	3	Y-Y	-	-
	3429	GAT - GAC	3	D-D	-	-
	3449	ATT - ACT	2	I-T	-	-
	3852	ATC - ATT	3	I-I	-	-
	4487	CTT - CCT	2	L-P	-	-
ycf2	549	TCG - TCA	3	S-S	-	-
	607	GAA - AAA	1	E-K	-	-
ycf3	44	TCT - TTT	2	S-F	+	+
	185	ACG - ATG	2	T-M	+	+
	191	CCA - CTA	2	P-L	+	+
	407	TCC - TTC	2	S-F	+	+
ycf4	254	TCA - TTA	2	S-L	-	+/−*

“+”: editing;

“-”: no editing;

“+/−”: partial editing;

“*”: editing sites shared with oil palm [3].

The editing types in coconut were all non-silent and 100% C-to-U. One occurrence of editing altered the initiator codon ACG to AUG in *ndhD* gene. Of these editing events, 62 (82.67%) occurred at the second base of the codon, 12 (16%) were at the first base of the codon, and only one (1.33%) was at the third base of the codon. The conversions of amino acids include 63 hydrophilic to hydrophobic (S to L, S to F, H to Y, T to M, R to W, T to I, and D to F), 11 hydrophobic to hydrophobic (P to L), and one hydrophobic to hydrophilic (P to S).

A comparative study of RNA editing across eight land plants demonstrated an evolutionary trend of decline (or complete loss) in the number of editing sites, silent editing, editing in the first or third position, and editing types other than C to U [47].

In angiosperms, the editing is almost exclusively a C to U substitution [49] and the total number of editing sites ranges from 20 to 37 [47], [50]–[53]. Compared with other angiosperms, coconut has more than twice as many editing sites, although the editing characteristics are similar (Table 7). Moreover, because of the evolutionary conservation of RNA editing, closely related taxa usually share more editing sites [47]. For example, more editing sites are shared within Poaceae than those shared among grasses and dicots [54]. Similarly, related *Nicotiana* species share more editing sites with each other than with plants from other genera [34].

**Table 7:** Comparison of RNA editing in six species of angiosperms.

	*Arabidopsis*	*Nicotiana*	*Cocos*	*Elaeis*	*Zea*	*Oryza*
Total editing sites	34	37	75	32	26	21
C to U editing (%)	100	100	100	78.12	100	100
U to C editing (%)	0	0	0	15.63	0	0
G to A editing (%)	0	0	0	6.25	0	0
Silent editing	0	0	0	10	1	0
Non-silent editing	34	37	75	18	25	21
Intron editing	0	0	0	4	0	0
1st codon editing (%)	14.7	5.4	16	15.4	4	4.8
2nd codon editing (%)	85.3	91.9	82.67	46.1	92	95.2
3rd codon editing (%)	0	2.7	1.33	23.5	4	0

### The rps19 pseudogenization and IR fluctuation

Dot plot analysis demonstrated that the gene content and organization of coconut cpDNA are nearly identical to other palm species (Fig. S1). Nevertheless, some variation could be detected. For instance, other palm species have two copies of the*rps19* gene located near the IR_A_/LSC and IR_B_/SSC junctions respectively, whereas coconut has only one copy of *rps19* at the IR_B_/SSC junction. At the IR_A_/LSC junction we found a *rps19*-like sequence of 174 bp, which is likely a pseudogene judged from its shorter length compared to the regular *rps19* gene (279 bp). We speculate that the pseudogenization of the *rps19* at IR_A_/LSC junction is due to IR fluctuation in coconut cpDNA.

**Figure 2:**
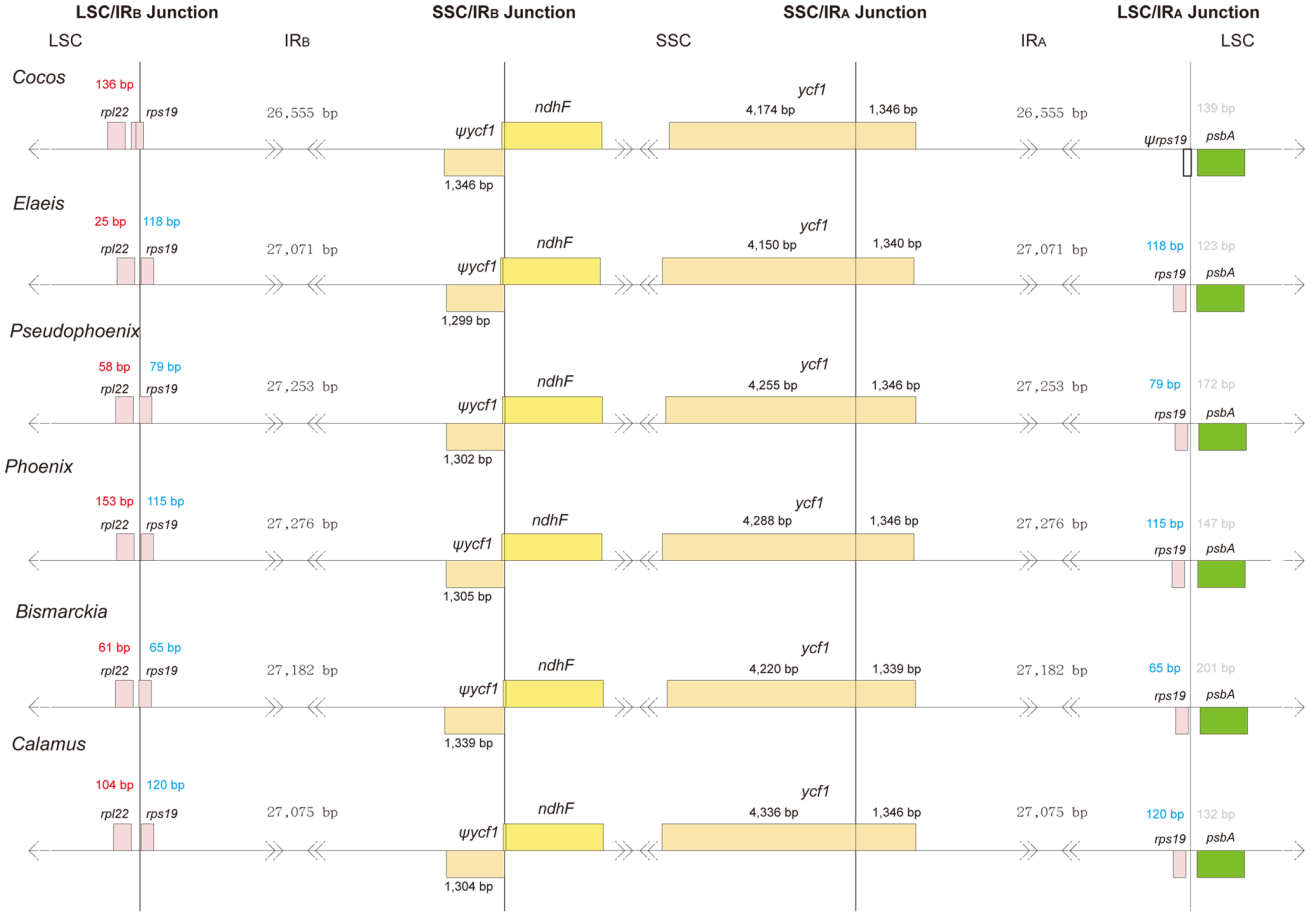
IR expansion into the LSC and SSC regions. Comparison of IR boundaries among six palm species. Numbers in red denote distance between *rpl22* and junction of LSC and IR_B_. Numbers in blue denote distance between *rps19* and junction of LSC and IR_A_. Numbers in gray denote distance between *psbA* and junction of LSC and IR_A_.

A comparative study among cpDNAs of six palm species (Table 5) indicated that coconut has the smallest cp genome (154,731 bp) and the shortest IRs (53,110 bp). The largest cp genome with the longest IRs is found in *Phoenix* (158,462 bp and 54,552 bp, respectively). Similarly to other cp genomes [2], [3], the palm cp genomes, including coconut, are all AT-rich. Graphical alignment showed that the IRs have both expanded and contracted during the evolution of the palm family, though dramatic changes were not detected (Fig. 2).

Fluctuations of the IR regions have occurred sporadically during the evolutionary history of angiosperms [55]. Two of the most extreme cases are found in *Pelargonium hortorum* of the Geraniaceae and a group of legumes that includes pea and broad beans. The single IR region has expanded to 76 kb [46] in the former whereas one copy of the IRs is completely lost from cp genomes of the latter [1]. The structurally conserved feature of the IR regions is resistant to recombinational loss [56]. The presence of the IR regions may thus help to stabilize the cp genome. The most direct evidence for this suggestion is that more rearrangements occurred within the group of legumes that have lost a copy of IR than those that have not [57]. Another piece of evidence is the acceleration of synonymous substitution rates in the remaining copy of the duplicated region [56]. Consequently, we can infer that the evolutionary rates of cp genomes in the palm family are relatively mild, judging from the comparatively minor fluctuation of the IR regions.

### Phylogenetic analysis and events of gene gain and loss

**Figure 3:**
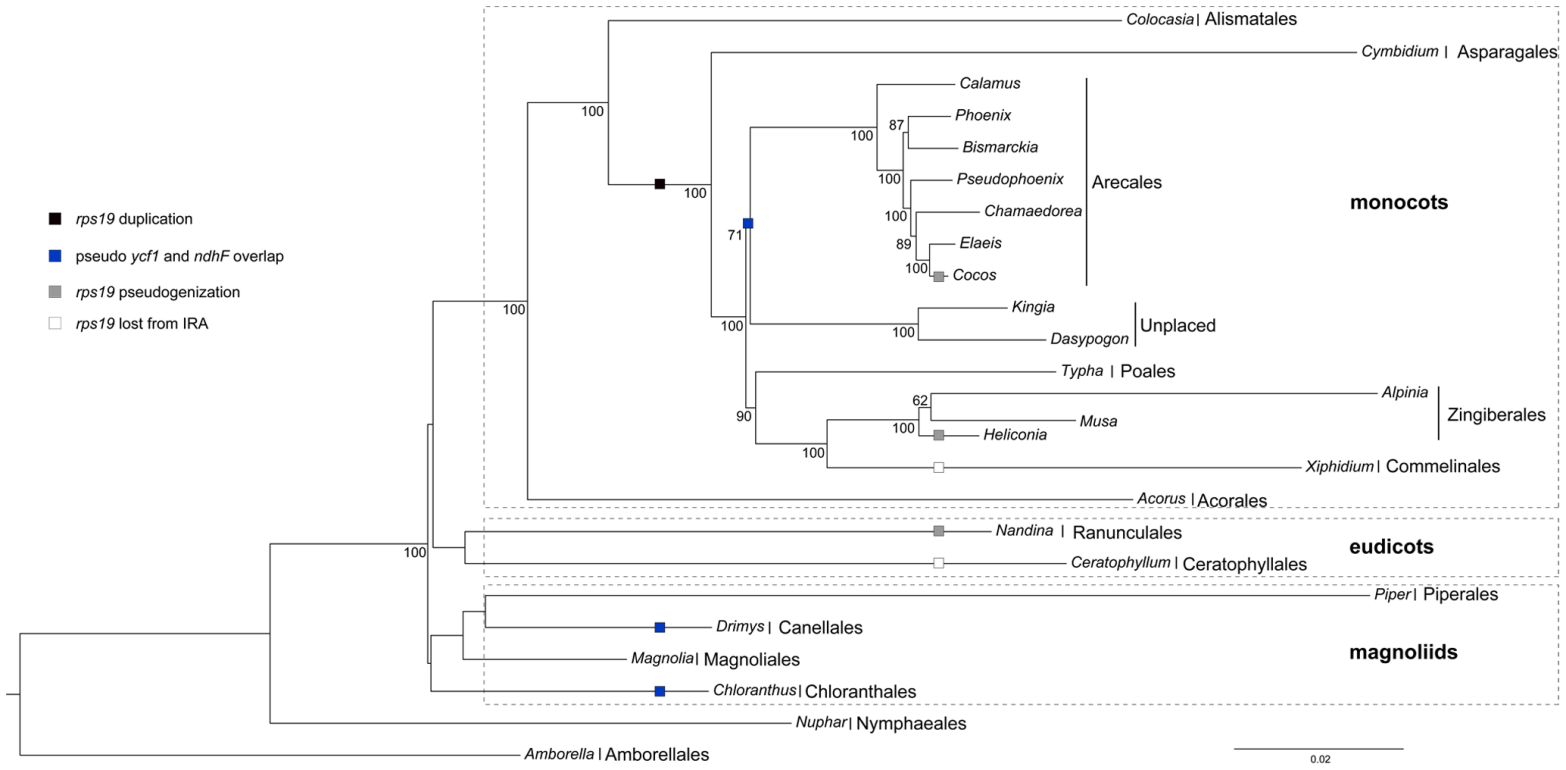
Phylogenetic tree of monocots. Numbers above/below the branches are bootstrap value (only values higher than 50% are shown). Black square denotes *rps19* duplication, gray square denotes *rps19* pseudogenization, white square denotes complete loss of duplicate *rps19*, and blue square denotes pseudo *ycf1* and *ndhF* overlap.

Our phylogenetic reconstruction built upon 47 protein-coding genes of cp sequences, rooted by *Amborella*, supported three major monophyletic groups: magnoliids, monocots, and eudicots (Fig. 3). Within monocots, *Acorus* (Acorales) diverged from other monocots first, followed by *Colocasia* (Alismatales), then by *Cymbidium* (Asparagales), which is sister to a clade that forms a monophyletic group of commelinids. The commelinids contain two sister clades. Within the first clade, Arecales group with the family Dasypogonaceae. In the second clade, Poales is sister to a subclade, which includes Zingiberales and Commelinales (Fig. 3). This topology is consistent with a phylogenetic study of commelinids based on 83 plastid genes [8]. Moreover, our inference of relationships within the Arecales is also congruent with a thorough study of the palm family using a supermatrix method with 16 data partition [58].

We then mapped the related gene duplication and pseudogenization events onto the tree according to parsimony criteria. Our results indicate that the duplication of *rps19* gene near the IR_A_/LSC junction likely occurred before the divergence of Asparagales from the remaining monocots, which consist of Arecales, a family (Dasypogonaceae) with indecisive order (*Dasypogon* and *Kingia*), Poales, Commelinales, and Zingiberales (Fig. 3). After the lineages differentiated, the duplicated *rps19* eventually became a pseudogene independently in *Cocos* of the Arecales, *Heliconia* of the Zingiberales, and *Nandina* of the Ranunculales. It has been completely lost in *Xiphidium* of the Commelinales and *Ceratophyllum* of the Ceratophyllales (Fig. 3).

In monocots, the overlap between *ndhF* and pseudo *ycf1* was found in a clade that contains Arecales and Dasypogonaceae. However, it was also found in *Drimys* of the Canellales and *Chloranthus* of the Chloranthales, both belong to the magnoliids. Following the parsimony rule, we concluded that the occurrence of the overlap between *ndhF* and pseudo *ycf1* in monocots and magnoliids arose from three independent events.

In summary, we have presented here the first complete cp genome sequence from coconut palm. Although the cp genome of coconut is the smallest found so far among palms, it shares the same overall organization, gene content and repeat structure that have been observed with cpDNA sequenced from other palm species. Nevertheless, unique features were found for the coconut genome, including pseudogenization of *rps19*-like gene and an unusually high number of RNA editing sites. A closer relationship between coconut and oil palms than with date palm was supported by phylogenetic relationships among angiosperms. Our data will contribute to the growing number of molecular and genomic resources available for studying coconut palm biology.

## Supporting Information

Figure S1 **Dot plot analysis.** The cp genomes are nearly identical in the palm family.
(TIF)

Table S1 Primers used for gap-filling PCR and RT-PCR.
(DOCM)
