# Supplementary figures and images for: Complete Sequence and Comparative Analysis of the Chloroplast Genome of Coconut Palm (Cocos nucifera)
Source: PLoS One. 2013 Aug 30;8(8):e74736. doi: 10.1371/journal.pone.0074736 (PMC3758300; doi:10.1371/journal.pone.0074736)

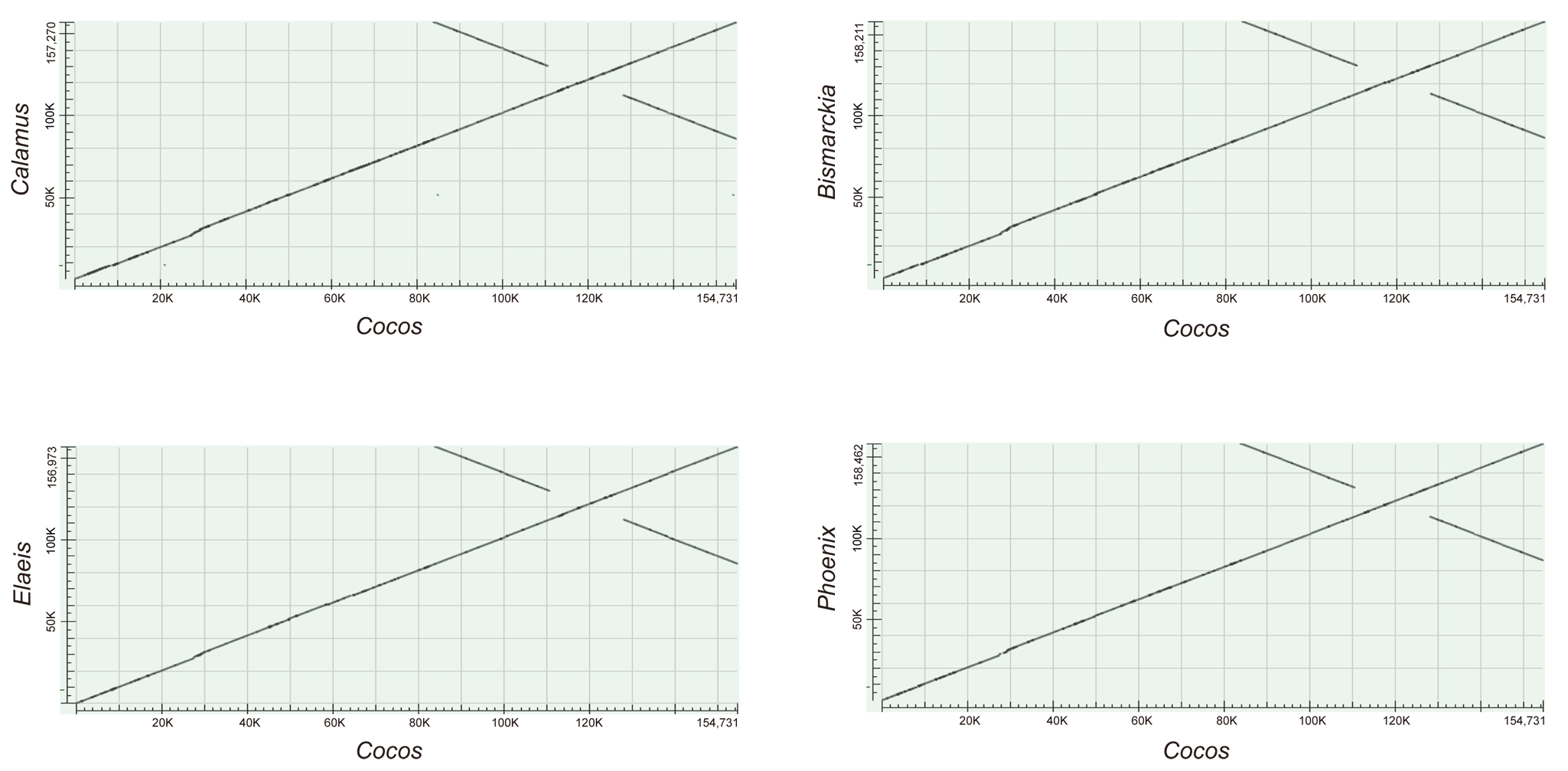

Supplement: Figure S1 — Dot plot analysis. The cp genomes are nearly identical in the palm family. (TIF) [file pone.0074736.s001.tif]
