# Supplementary material for: Complete Sequence and Comparative Analysis of the Chloroplast Genome of Coconut Palm (Cocos nucifera)
Source: PLoS One. 2013 Aug 30;8(8):e74736. doi: 10.1371/journal.pone.0074736 (PMC3758300; doi:10.1371/journal.pone.0074736)
Supplement: Table S1 — Primers used for gap-filling PCR and RT-PCR. (DOCM) [file pone.0074736.s002.doc]

Supplementary Table S1. Primers for gap-filling PCR and RT-PCR.

| Gene | Direction | Primer sequences (5' to 3') |
| --- | --- | --- |
| accD | F | GGGCAAGAAGACTCTATGGAAAAATG |
| accD | RT | CAAGGGAAAGAAACCGTGGAGCTG |
| atpA | F | CATTATTTTTCCCCTTTGCTTCCG |
| atpA | RT | GCTTGCTCGGTGAATGTCTTGGTAG |
| atpB | F | CAACTTGCTATCGGACATTTATTTTCG |
| atpB | RT | GGTAATACGCCAATTTGGCCAC |
| atpB | F | CTTCCCGAGCAGGCCTTTTATTTG |
| atpF | RT | GGACGACGAAGTGATACAAAAAGAAC |
| atpF | F | GCAGTCGGATGGATCCAACC |
| atpI | RT | CGATATATCGGGTGTAGAAGTAGGCC |
| atpI | F | CCGCCGTCTATCTTTTTTCCTACG |
| ccsA | RT | GTGGCGGCATCCTCTAAAAGAG |
| ccsA | F2 | GTAATGAGATGCTTGTTCGAGTTTTTG |
| ccsA | RT2 | CGTAGGATTACGTGATTCATCGG |
| ccsA | F | GTGCCGAATTCGTACCTTGTAAAC |
| clpP | RT | GGAGAGGAAGATGCAGTTTGGG |
| clpP | RT | CCTAGTATTTTCATTTTCGACCGC |
| matK | F | CATAAGTCTCATCACGTCACCAAACC |
| matK | F | GGGTCCTGTCTCTGGTTCAAGTAG |
| ndhA | RT | CATTATGGGAGAAGTTGATCGTTG |
| ndhA | F | GAGGTTATGAATCTGGAATATCGTTATC |
| ndhB | RT | GGGACTTTTTCGGAGATTGGATGC |
| ndhB | F | CTAAAAGCTAAAAGAGGGTATCCTGAG |
| ndhB | RT | GGAGCCGTGCGAGATGAAAGTC |
| ndhB | F | GACTGGACGAAACCAAGAAATAACC |
| ndhB | RT | CATCGGAACAATAGGGCCG |
| ndhC-trnV | F | CGCATAGTTTAGAGTTTGTTTGCTG |
| ndhD | RT | CGAGCACGGGTTTTTCTGGTC |
| ndhD | F | CGAGAGAACGAAATCAGGATAAATACC |
| ndhD | RT | GGTATTTATCCTGATTTCGTTCTCTCG |
| ndhD | F | CAGAATGCATAGGTTGTTAGAAGGAG |
| ndhF | RT | GAGATAAGAATTGGTGAATCGGAAAC |
| ndhF | F | CGGAAATGATAGAACGAAAAATGTC |
| ndhF | RT | GCGGCTTTATTCCATTTGATCACTC |
| ndhF | F | GTACTATTTACTTTGTTCGTTGG |
| ndhG | RT | GATCAGTTCTTGTATTAGGAGGTCTGG |
| ndhG | F | GACGAGCCATAGTAATTGCACC |
| ndhH | RT | CAACGATCAACTTCTCCCATAATG |
| ndhH | F | GCCCTCACCACCCATCAATG |
| ndhK | RT | GGAGCCATTTTCATTGTTACTGTGCC |
| ndhK | F | GTTGGTTCAGTTTATGCATGGCG |
| ndhK | RT | GACCCGCTGTTTCTTATTCTGC |
| petB | F | GTGAGATTTATTTGTTTCGGTATTTCC |
| petB | RT | GATCTGTCTTCTCTATAAAGGACCCG |
| petN | F | GTCCACTTCTTCCCCATACTACGAG |
| psaI | RT | GAAATATTAAATCGAGGCACCCATTC |
| psaI | F | GATGAGATTTGGTCCCATCGG |
| psbA | RT | CTTACATTTCTTCTTGGCTGCTTGGC |
| psbA-rps19 | F | GGAGCAATACCCAACTAAAACAAG |
| psbD | RT | CCTGTAAACCAACCTCCTAAAGCG |
| rpl2 | F | GCAAAATACTTAATAACACGGCGATAC |
| rpl2 | RT | CCGCACTCAAAGGTAGGGC |
| rpl2 | R | CGGCGACGAAGAATAAAACTATCAC |
| rpl20 | F | GATTCAATGACCAGAGTTAGACGAGG |
| rpl20 | R | GGAATTCTGTTTCAATTTCAATCATTTC |
| rpl22 | F | GCACTCCTATTGACTTCGGCTTTAC |
| rpl22 | F | CATTAGTGGGGGATGACCTTATG |
| rpl23 | R | CCCGGTTGAAGCGTAATGATC |
| rpoA | R | GTATGGTTCGAGAGGAAGTAGTAGGAT |
| rpoA | F1 | CCAATATCTGTTTTACATCTTCTATTCG |
| rpoB | F2 | GATGCTCCGGAATGGAAATGAGG |
| rpoB | R | GCATCCTCGGGGTTAGGTACTCTTC |
| rpoB | R | CAGTCTCCGGTTCATATTTCGTCG |
| rpoB | F | GACATTCCCTCATTTCCATTCCGG |
| rpoB | F | GGACGGTGCGGCTACAGTTGG |
| rpoC1 | R | CGACCGGTATAAACATCAACAACTTCG |
| rpoC1 | F | GGGCACGGGCATCACCTAATC |
| rpoC1 | R2 | CCATCTTTTTCCGGTTTATTGG |
| rpoC2 | F | CTTATGGCAGAACGGGCTGATC |
| rpoC2 | R | CTTGCCCTATCATACCTTTTTCCG |
| rpoC2 | R | CATTTGATCTTGATCTTTGTGGAGCG |
| rps14 | F | GGAACTGAACATTATGGCAAGAAAAAG |
| rps14 | F | GTCCAGATAGCCCAAAGTCTCGATAG |
| rps15 | R | GACAAATAAGCCAGGAGCCG |
| rps19 | R2 | GGTATAATGGTAGATGCCCGAGAC |
| rps2 | R2 | CGAAATATCTGCGAGATCTGGGTC |
| rps2 | R3 | GGAAGAGATGATGGAAGCAGGAG |
| rps3 | F2 | GGAAAGAGAACATAATAGAAAAATATGGG |
| rps3 | R | CAGAATGGCAAGACAAATGAAGG |
| rps7 | F | GAGTCTCTTCCTTTTTGCGTATGG |
| rps7 | RT | CATGTCACGTCGAGGTACTGCAG |
| rps8 | RT2 | GTTATGGGTAGGGACACTATTGCCG |
| rps8 | RT | CCAGAGGAGGATCACCATATATAACAC |
| rrn4.5 | F | CTAACAGACCGAGAGATTTGAACCTTG |
| trnM-rpl23 | RT | GGCTCTGTATCAATGGAATCTCATC |
| trnM-trnV | F | CTGTTAACAGTAGACGGATCAGAGAG |
| ycf1 | F2 | GTTGTTGCTGATACCTCCTTCTCGG |
| ycf1 | RT | CCGAGAAGGAGGTATCAGCAACAAC |
| ycf1 | F | GGCAGTGGATTCATTGACGAG |
| ycf1 | RT | GCATGGTCCGCTCAATAAAGG |
| ycf1 | F | CGGAAATGATAGAACGAAAAATGTC |
| ycf1 | RT | CCTTGACATACTGAAACGACTGCC |
| ycf1 | F | CAATCAGGAATCAAATAGAACTAGAACG |
| ycf1 | RT | CCTGTTGTTCGTTTTTTTCTTGG |
| ycf15 | F2 | CCAGTTGACCGAGCCTAATTC |
| ycf2 | F2 | CCATGCAGTACCAGACACGAGATAG |
| ycf2 | F3 | GATTATGTTTCGCTTCTTCCTCGG |
| ycf2 | R | CATCGTACATCGTGAATAACCAAATTC |
| ycf3 | R | CGACCGTAACGGATGTTGGCTC |
| ycf3 | R | GTGGTCTTATTCGAATTCGAAACGC |
| ycf3 | F2 | GATTGCTAATTATGCCTAGATCTCGG |
| ycf4 | F3 | CTGGATCTAGTAGAGTATGAATTGGCG |
| ycf4 | R4 | GGTACGCGCAAGAAATAGGCC |
